# Neuroprotective Effect of Stearidonic Acid on Amyloid β-Induced Neurotoxicity in Rat Hippocampal Cells

**DOI:** 10.3390/antiox11122357

**Published:** 2022-11-28

**Authors:** Yueru Li, Wencong Lai, Chen Zheng, Jeganathan Ramesh Babu, Changhu Xue, Qinghui Ai, Kevin W. Huggins

**Affiliations:** 1 Key Laboratory of Aquaculture Nutrition and Feed, Ministry of Agriculture, Ocean University of China, 5 Yushan Road, Qingdao 266005, China; 2 Department of Nutrition, Dietetics and Hospitality Management, Auburn University, Auburn, AL 36849, USA; 3 College of Food Science and Engineering, Ocean University of China, Qingdao 266005, China; 4 Boshell Diabetes and Metabolic Diseases Research Program, Auburn University, Auburn, AL 36849, USA

**Keywords:** Alzheimer’s disease, stearidonic acid, omega-3 fatty acids, hippocampal cells, neuroprotective

## Abstract

Dietary intake of omega-3 fatty acids found in fish has been reported to reduce the risk of Alzheimer’s Disease (AD). Stearidonic acid (SDA), a plant-based omega-3 fatty acid, has been targeted as a potential surrogate for fish-based fatty acids. However, its role in neuronal degeneration is unknown. This study was designed to evaluate effects of SDA on Amyloid-β(A-β)-induced neurotoxicity in rat hippocampal cells. Results showed that SDA effectively converted to eicosapentaenoic acid (EPA) in hippocampal cells. Aβ-induced apoptosis in H19-7 cells was protected by SDA pretreatment as evidenced by its regulation on the expression of relevant pro- and anti-apoptotic genes, as well as the inhibition on caspase activation. SDA also protected H19-7 cells from Aβ-induced oxidative stress by regulating the expression of relevant pro- and anti-oxidative genes, as well as the improvement in activity of catalase. As for Aβ/LPS-induced neuronal inflammation, SDA pretreatment reduced the release of IL-1β and TNFα. Further, we found that the anti-Aβ effect of SDA involves its inhibition on the expression of amyloid precursor protein and the regulation on MAPK signaling. These results demonstrated that SDAs have neuroprotective effect in Aβ-induced H19-7 hippocampal cells. This beneficial effect of SDA was attributed to its antiapoptotic, antioxidant, and anti-inflammatory properties.

## 1. Introduction

Alzheimer’s disease (AD) is an emerging public health concern and one of the leading causes of death for the global aging population [1]. Despite progress in symptomatic therapy for AD, effective therapeutic approaches that interfere with AD are still unavailable [2]. AD is most associated with aging, but also largely affected by dietary nutrition [3]. It is thus essential to identify the nutritional biological factors that could modulate the AD progression. Increased consumption of ω-3 polyunsaturated fatty acids (ω-3 PUFA), mainly EPA (20:5; ω-3) and DHA (22:6; ω-3), which have been reported to be associated with reduced risk of AD [4]. Cold water fish and fish oils are the most direct source of DHA and EPA. However, many individuals cannot tolerate the taste or smell of oily fish or fish oils, even when provided in capsules [5]. In addition, yields from global fisheries have been reported to be stagnant or declining [6]. In addition, there is an increasing alarm over levels of accumulated contaminant in some species of long-lived fish [7]. Hence, there is a need and desire to identify alternative sources of DHA and EPA that have similar biological properties.

**Figure 1:**
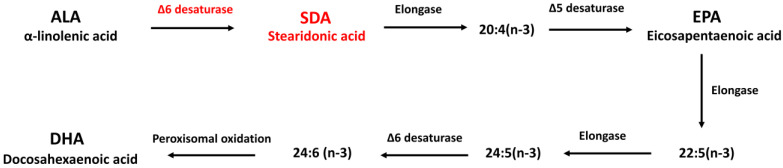
Metabolism of essential ω-3 fatty acids.

Alpha-linolenic acid (ALA; 18:3; ω-3) is the major ω-3 fatty acid source available in vegetable oils such as flaxseed oil, canola oil, or soybean oil. However, the conversion from ingested ALA to DHA and EPA is limited due to a rate-limiting step in ω-3 fatty acid metabolism catalyzed by Δ6-desaturase [8] (Figure 1). Stearidonic acid (SDA; 18:4; ω-3), as a metabolic intermediate between ALA and EPA, represents the Δ6 desaturation product of ALA, and thus bypasses the rate-limiting step in the conversion of dietary ALA to DHA and EPA [9]. Due to its relatively efficient conversion following consumption, SDA has been targeted as a potential biologically active surrogate for EPA [10]. Studies have shown that consumption of SDA as ethyl esters, echium oil, or SDA soybean oil increased EPA levels in red blood cells [11], peripheral blood mononuclear cell [12], neutrophils [13], and 3T3-L1 embryo fibroblasts [9]. Feeding with SDA increased the EPA content in many tissues of rodents including brain [14,15]. The efficacy of SDA on EPA enrichment in different tissues ranges from 17% to 85%, as much as the efficacy of EPA on EPA enrichment based on human studies [9,11,12,15,16,17], while the conversion of ALA to EPA is less than 7% in humans [18]. Additional studies have shown that SDA was able to improve lipid profile [12], attenuate hepatic steatosis [19], reduce atherosclerosis [20], decrease leukotriene generation [21], inhibit inflammation [22] and slow cancer growth [23]. In addition, in a recent study by Kotlega and colleagues [24], serum SDA levels are positively correlated with the cognitive functions in stroke survivors, suggesting SDA could be a new supplemental source of long-chain ω-3 PUFAs in health promotion and disease prevention.

AD is a major chronic neurodegenerative disorder characterized by progressive neuronal death, loss of memory and impairment of higher cognitive functions [25]. The neuropathological hallmarks of human AD brain are the presence of extracellular plaques composed of amyloid β (Aβ) and intracellular neurofibrillary tangles composed of hyperphosphorylated tau protein [26]. Plaques and tangles modulate oxidative injury, inflammatory responses, and cell apoptosis [27]. The exact etiology of AD is unknown but overproduction of Aβ, exaggerated oxidative stress, and neuroinflammation are widely recognized in individuals with AD and thereby play important roles in modulating neuronal death [28]. Therefore, Aβ has been used as an inducer to mimic AD and develop effective drugs and therapies in many studies [29]. In addition, the hippocampus is the center of cognitive function in the brain and is vulnerable to damage early in the development of AD. Therefore, H19-7 hippocampal cells have been used extensively as a cell culture model to study the molecular control of AD [25]. Recent studies highlighted the beneficial effect of long-chain polyunsaturated ω-3 fatty acids (EPA and DHA) in AD which may be attributed to their antioxidant, anti-inflammatory, antiapoptotic and neurotrophic properties [26]. However, the effect of SDA on neurotoxicity is unknown.

Therefore, this study aimed to investigate the effect of SDA on Aβ-induced neurotoxicity in H19-7 rat hippocampal cells. First of all, SDA effectively converted to EPA in hippocampal cells (about 10%, 2.6-fold than ALA). In addition, SDA protected Aβ-induced oxidative stress, inflammation and apoptosis by regulating the expression of relative genes, changing the activity of relative enzymes, and affecting phosphorylation of mitogen-activated-protein kinase (MAPK) pathways. These results support the use of SDA, an effective surrogate for EPA, as dietary supplement for AD.

## 2. Materials and Methods

### 2.1. Cell Culture

The H19-7 cell line was derived from hippocampi dissected from embryonic day 17 (E17) Holtzman rat embryos and immortalized by retroviral transduction of temperature sensitive tsA58 SV40 large T antigen. The cells were generously provided by Dr. Ramesh Jeganathan. All cells were cultured in poly-D-lysine-coated culture dishes and were maintained in Dulbecco’s modified Eagle’s medium (DMEM, Gibco, New York, NY, USA) supplemented with 10% fetal bovine serum (FBS, Invitrogen, Waltham, MA, USA), 1% penicillin–streptomycin (Sigma, St. Louis, MO, USA), 0.001 mg/mL puromycin (Sigma, St. Louis, MO, USA), and 0.2 mg/mL G418 (Sigma, St. Louis, MO, USA) in a humidified incubator at 34 °C with 5% CO_2_.

### 2.2. Fatty Acid Treatment

Fatty acids (ALA, SDA, DHA and EPA) were purchased from Matreya LLC, (Stage College, PA, USA). Stock solutions of ω-3 fatty acids were placed in ethanol and further pre-incubated at 34 °C for 1 h in DMEM containing 1.5% of fatty acid-free bovine serum albumin (BSA, Thermo Fisher Scientific, Waltham, MA, USA) to allow albumin conjugation. After incubation at 34 °C for 1 h, fatty acid-supplemented medium (100 μM) or BSA–ethanol vehicle control was applied to H19-7 hippocampal cells for 2 days. Fatty acids were delivered to the cells as fatty acid/BSA complexes. BSA–ethanol vehicle was used as control. For treatment analysis, cultured H19-7 cells were pretreated with ω-3 fatty acids followed by exposure to 30 μM Aβ1-40 peptide for 24 h. Aβ1-40 was supplied by Sigma Chemical Co. (St. Louis, MO, USA) and dissolved in deionized distilled water at a concentration of 1 mM and stored at −20 °C until use. The stock solutions were diluted to the desired concentrations and pre-incubated at 34 °C for 4 days prior to experiments to allow aggregation. After pretreatment with ω-3 fatty acids, Aβ1-40 in serum-free medium was added to the H19-7 cells. Based on previous studies, 30 μM of Aβ1-40 peptide was chosen to induce neurotoxicity including oxidative stress, inflammation and apoptosis [30]. For Aβ-induced neuroinflammation, 100 ng/mL LPS (Sigma, St. Louis, MO, USA) was added together with Aβ1-40. After treatment, cells were lysed with RIPA lysis buffer.

### 2.3. Analysis of EPA and DHA Content

H19-7 hippocampal cells incubated with fatty acids for 48 h were used for fatty acid analysis. Lipid extracts from H19-7 cells were prepared using chloroform/methanol (C/M, 1/1, *v*/*v*). The organic phase was collected, dried under N2 gas, and dissolved in C/M 1/1. Saponification and formation of fatty acid methyl esters comprising cellular lipid was then performed for liquid chromatography/mass spectrometry (LC/MS). The instrument we used is Agilent 1290 UHPLC coupled Agilent 6460 QQQ triple quadruple mass spectrometer. LC/MS was conducted to quantify the content of DHA and EPA within cells. Palmitic acid-d31 (Sigma, St. Louis, MO, USA purity > 99%) was added as internal standard. Fatty acid content was normalized to the protein content. Protein quantification was performed using the Bio-Rad DC Protein Assay Kit (Bio-Rad, Hercules, CA, USA). BSA standard curve and sample preparation and analysis were realized according to manufacturer’s instructions.

### 2.4. MTT Assay

The MTT staining method was conducted as previously described [31]. Briefly, the H19-7 hippocampal cells were seeded in 96-well plates at a density of 2 × 10^4^ cells/mL/well. In total, 100 μM different ω-3 fatty acids were added to the H19-7 cells for 48 or 96 h. For Aβ-induced apoptosis, H19-7 cells were pretreated with 100 μM different ω-3 fatty acids for 48 h and Aβ1-40 was then added to the cells for another 24 h incubation. At the end of the treatment, the culture medium was removed and replaced with sterile-filtered 3-(4,5-dimethylthiazol-2-thiazolyl)-2,5 diphenyl-2H-tetrazolium bromide (MTT, Sigma Aldrich, St. Louis, MO, USA) solution. After further incubation with MTT solution at 37 °C for 4 h, the medium was aspirated, allowed to dry completely. Thereafter, 200 μL of dimethyl sulfoxide (DMSO, Sigma Aldrich, St. Louis, MO, USA) was added to each well. The microtiter plate was placed on a shaker in order to dissolve the dye. After the formazan crystals had dissolved, the absorbance was determined spectrophotometrically at 490 nm using a reference wavelength of 630 nm on the Bio-Tek spectrophotometer (Winooski, VT, USA).

### 2.5. Total RNA Isolation and Quantitative Real-Time PCR (qRT-PCR) Analysis

H19-7 cells incubated within different ω-3 fatty acids (100 μM) for 48 h followed by 24 h induction with Aβ1-40 were washed with PBS and total RNA was extracted using RNeasy Mini Kit (Qiagen; Valencia, CA, USA) according to manufacturer’s instructions. The quality and concentration of total RNA was determined spectrophotometrically using NanoDrop (Thermo Fisher Scientific, Waltham, MA, USA). Complementary DNA (cDNA) was synthesized from 1μg of RNA using iScriptTM cDNA Synthesis Kit (Bio-Rad, Hercules, CA, USA) according to the manufacturer’s protocol. Reverse transcription was performed with sample incubation at 25 °C for 5 min, followed by 42 °C for 30 min and then 85 °C for 5 min. The synthesized cDNA was used immediately for real-time PCR or stored in a −20 °C freezer. Quantitative real-time PCR was performed in the MyiQ single-color real-time PCR detection thermocycler (Bio-Rad, Hercules, CA, USA) using iQTM SYBR^®^ Green Supermix (Bio-Rad, Hercules, CA, USA) to evaluate gene expression. Rat gene-specific primers were designed from Primer Bank and constructed by Integrated DNA Technologies, Inc. (IDT, Inc., Coralville, IA, USA). Oligonucleotide sequences of the primers used for amplification are presented in Table 1. Reaction mixtures were incubated for an initial denaturation at 95 °C for 3 min followed by 40 cycles of 95 °C for 30 s, 60 °C for 30 s, and 55 °C for 10 s. The cycle threshold (ΔCT) method was used to measure relative quantification of the target gene, where values were normalized to the reference gene, β-actin. Fold changes of gene expression were calculated by the 2^−ΔΔCT^ method. The statistical analysis was based on ΔCT values.

### 2.6. Western Blot Analysis

The H19-7 cells were washed with the ice-cold PBS buffer and harvested from the culture plate with cell lysis buffer (RIPA, Thermo Fisher Scientific, Waltham, MA, USA) containing protease inhibitor cocktail (Thermo Fisher Scientific, Waltham, MA, USA). The cell lysate was centrifuged at 10,000× *g* at 4 °C for 15 min to remove the insoluble material. The protein concentrations were estimated with the Bio-Rad DC Protein Assay Reagent using BSA as a standard. The proteins mixed with sample loading buffer were boiled at 95 °C for 5 min and then separated in 10% sodium dodecyl sulfate polyacrylamide (SDS-PAGE) gel. The proteins in the gel were transferred onto polyvinylidene fluoride (PVDF) membranes (Millipore, Temecula, CA, USA). The membrane was blocked in 5% non-fat dry milk in the Tris Buffered Saline (TBS) with 0.1% Tween-20. The blocked membrane was incubated with appropriate primary antibodies, and then corresponding secondary antibodies. The membrane was developed using an enhanced chemiluminescent substrate (GE Healthcare, Piscataway, NJ, USA).

### 2.7. Total Antioxidant Capacity (T-AOC) Assay

To measure total antioxidant capacity of H19-7 cells affected by ω-3 fatty acids with Aβ1-40 induction, cells were pretreated with different ω-3 fatty acids (100 μM) for 48 h followed by 24 h induction with Aβ1-40. After washing with PBS, the total antioxidant potential of samples was determined spectrophotometrically at 570 nm by using a Total antioxidant capacity assay kit (Abcam, Cambridge, UK) according to manufacturer’s instructions. These kit measures combined nonenzymatic antioxidant capacity. Briefly, both small molecules and proteins that carry anti-oxidant capacity are able to convert Cu^2+^ ion to Cu^+^ ion. The reduced Cu^+^ ion is chelated with a colorimetric probe that will display a broad absorbance peak around 570 nm, proportional to the total antioxidant capacity. A standard concentration of 6-hydroxy-2,5,7,8-tetramethylchroman-2-carboxylic acid (Trolox) was used to create a calibration curve and the results of the assay were expressed as nanomoles per microliter Trolox equivalents. Values were normalized to the protein content.

### 2.8. Catalase Activity Assay

To measure the activity of anti-oxidant enzyme catalase, H19-7 cells treated with different ω-3 fatty acids and induced with Aβ1-40 were collected. After washing with PBS, the catalase enzyme activity of samples was analyzed spectrophotometrically at 570 nm by using a Catalase Assay Kit (Abcam, Cambridge, MA, USA) according to manufacturer’s instructions. Briefly, catalase first reacts with H_2_O_2_ to produce water and oxygen. Thereafter, the unconverted H_2_O_2_ will react with OxiRed probe to formulate a product which can be measured at 570 nm. A standard concentration of hydrogen peroxide was used to create a calibration curve and the results of the assay were expressed as microunits per microgram protein.

### 2.9. Enzyme-Linked Immunosorbent Assay (ELISA)

The levels of proinflammatory cytokines (IL-1β, IL-6, and TNFα) in the H19-7 hippocampal cells pretreated with different ω-3 fatty acids and induced by Aβ1-40 and LPS were determined with Quantikine ELISA kits (R&D Systems, Minneapolis, MN, USA) according to the manufacturer’s instructions. Briefly, H19-7 cells were pretreated with 100 μM of ALA, DHA, EPA, or SDA for two days, followed by 24 h incubation with Aβ1-40 and LPS. At the end of the treatment, cell culture supernatant was collected into a centrifuge tube and centrifuged at 10,000× *g* for 15 min at 4 °C. The centrifuged supernatant was then ultracentrifuged at 150,000× *g* for 2 h at 4 °C in a vacuum centrifuge. The ultracentrifuged supernatant samples were immediately stored at −80 °C until use. Bio-Rad DC Protein Assay (Bio-Rad, Hercules, CA, USA) of the cells was performed for each sample. The supernatant was used in ELISA. The quantity of IL-1β, IL-6, and TNFα in each sample was standardized to its corresponding protein contents.

### 2.10. Statistical Analysis

All data are presented as mean ±SEM. The statistical significance of differences between groups was determined by one-way analysis of variance (One-way ANOVA) and Student’s *t*-test (two-tailed). The results were considered to be significant when the value of *p* was < 0.05. Figures were produced by GraphPad PrismTM version 6.01 (GraphPad software, San Diego, CA, USA).

## 3. Results

### 3.1. SDA Effectively Converted to EPA in Rat Hippocampal Cells

**Figure 2:**
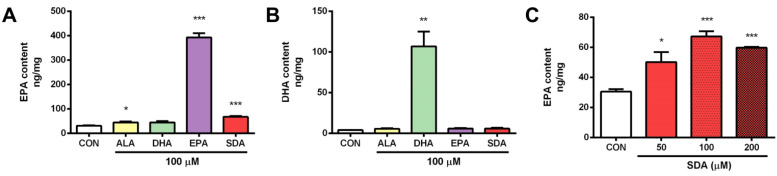
SDA effectively converted to EPA in rat hippocampal cells. H19-7 hippocampal cells were treated with ALA, DHA, EPA, SDA, or BSA–ethanol vehicle for 48 h. (**A**) EPA enrichment by ethanol vehicle control or 100 μM of ALA, SDA, EPA, and DHA in H19-7 hippocampal cells. (**B**) DHA enrichment by ethanol vehicle control or 100 μM of ALA, SDA, EPA, and DHA in H19-7 hippocampal cells. (**C**) EPA enrichment by SDA (0, 50, 100, and 200 μM) in H19-7 hippocampal cells. Values were obtained from three independent experiments and were expressed as the means ± SEM. Data were normalized to the protein contents; * *p* < 0.05, ** *p* < 0.01, *** *p* < 0.001, different from BSA–ethanol vehicle-treated control cells.

To test the conversion of SDA to EPA and DHA in hippocampal cells, H19-7 were cultured in the presence of SDA (100 μM). After 2 days, the cellular content of EPA was increased. As shown in Figure 2A, except DHA, both ALA and SDA significantly increased cellular EPA content, but with different extent, that is ALA 1.46-fold and SDA 2.21-fold compared to control group. As we expected, the conversion efficacy of SDA is higher than that of ALA. However, compared to EPA, which led to a 12.9-fold increase in cellular EPA content, SDA was approximately 17% as effective as EPA in hippocampal cells (Figure 2A). As for the conversion to DHA, none of the precursors (ALA, SDA and EPA) could increase the cellular DHA content; only DHA incubation itself led to a 26.4-fold increase in hippocampal cells (Figure 2B). To confirm the conversion of SDA to EPA, we examined the cellular EPA content in H19-7 cells cultured with different concentrations of SDA. As shown in Figure 2C, 50 μM of SDA incubation was enough to induce a significant change in EPA levels, while 200 μM of SDA was not better than 100 μM in EPA enrichment, indicating that the conversion of SDA to EPA is limited by the metabolic enzymes (elongase and Δ5-desaturase, Figure 1) when there were sufficient available substrates. These results verified that SDA can be a surrogate for EPA.

### 3.2. SDA Protects against Aβ-Induced Hippocampal Cell Death

**Figure 3:**
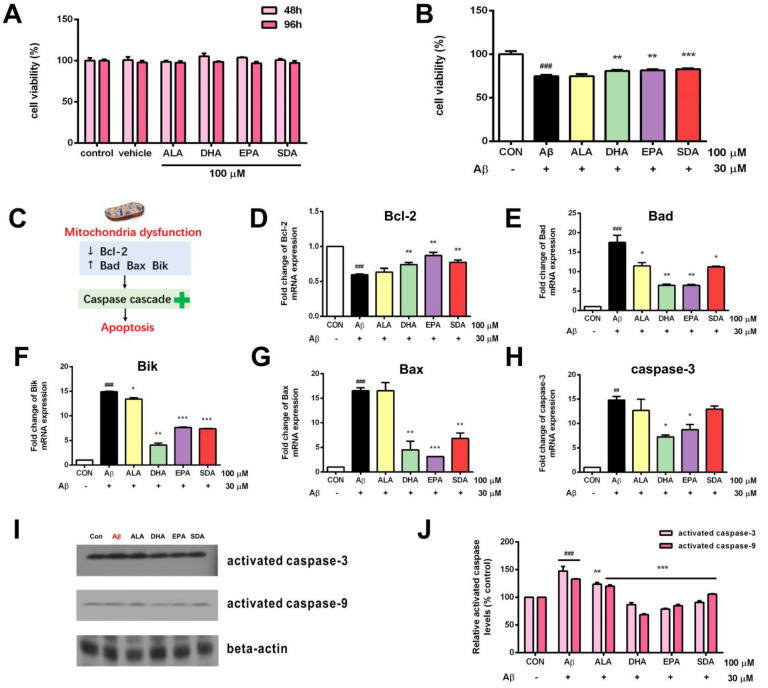
SDA protects against Aβ -induced hippocampal cell death. (**A**) The viability of H19-7 cells after treatment with ALA, DHA, EPA, SDA, or BSA–ethanol (vehicle) in the concentration of 100 μM for 48 h or 96 h. (**B**) The viability of H19-7 cells after 48 h of culture in medium enriched with ALA, DHA, EPA, SDA prior to exposure to non-fibrillar Aβ for 24 h. (**C**) Aβ strongly decreased anti-apoptotic gene Bcl-2 expression and increased the expression of pro-apoptotic genes, caspase-3, Bad, Bax, and Bik. H19-7 cells were pretreated with ALA, DHA, EPA, SDA, or BS–-ethanol vehicle control in the concentration of 100 μM for 48 h, then 30 μM Aβ1-40 was added into the medium for another 24 h incubation. The mRNA expression of anti-apoptotic gene (**D**) Bcl-2 and pro-apoptotic gene (**E**) Bad, (**F**) Bik, (**G**) Bax, and (**H**) caspase-3 in H19-7 cells. (**I**,**J**) The protein expression of the activated caspase-3 and activated caspase-9 in H19-7 cells. Values were obtained from three independent experiments and were expressed as the means ± SEM; ## *p* < 0.01, ### *p* < 0.001, different from BSA–ethanol vehicle-treated control cells; * *p* < 0.05, ** *p* < 0.01, *** *p* < 0.001, different from Aβ1-40-treated cells.

Although ω-3 fatty acids are toxic-free, we still first confirmed that the treatment condition (concentration and duration) of ω-3 fatty acids which we planned to use in our experiments is safe to hippocampal cells. As shown in Figure 3A, the incubation with 100 µM of ω-3 fatty acids (ALA, SDA, EPA and DHA) for 2, even 4 days, had no effect on the cell viability of H19-7 cells. Therefore, all the following experiments were done with 100 µM of ω-3 fatty acids for 2 days of pre-incubation plus on the 3rd day of Aβ-induction. Aβ40 is the predominant C-terminal variant of the Aβ protein constituting the majority of Aβs. It undergoes post-secretory aggregation and deposition in the AD brain. Rege et al. used Aβ40 (25 µM, 24 h) to induce oxidative stress and tau phosphorylation in H19-7 cells to mimic AD-like damages [29]. In the present study, H19-7 cells treated with Aβ40 (30 µM, 24 h) exhibited increased cell death (Figure 3B). Cell apoptosis dysregulation is mediated by mitochondrial dysfunction, which can be characterized by the expression of pro-apoptotic (Bax, Bad, Bid) and anti-apoptotic (Bcl-2) members (Figure 3C). Mitochondria dysfunction is critical regulator of cell death, a key feature of neurodegeneration. Mitochondrial dysfunction also includes the release of cytochrome c, which activates a downstream caspase cascade (Figure 3C). Activated caspases can also affect the function of mitochondria. Therefore, we next measured these apoptotic markers. As we expected, the expression of anti-apoptotic gene Bcl-2 was decreased in the Aβ-induced group when compared to the control group (Figure 3D). The expression of pro-apoptotic genes including Bad (Figure 3E), Bik (Figure 3F), Bax (Figure 3G), caspase-3 (Figure 3H) and the activation of caspases including caspase-3 and caspase-9 (Figure 3I,J) were increased in the Aβ-induced group when compared to the control group. However, ω-3 fatty acid pretreatment protected against cell death (Figure 3B), up-regulated the anti-apoptotic Bcl-2 gene expression (Figure 3D), inhibited the expression of pro-apoptotic genes (Figure 3E–H), and attenuated the activation of caspases (Figure 3I,J) in Aβ-induced H19-7 cells. Interestingly, this protective effect of SDA on Aβ-induced neurotoxicity was much comparable to the effect of EPA and DHA, indicating that the plant-sourced SDA might be a surrogate for fish-sourced ω-3 fatty acids since it has the same beneficial impact as EPA and DHA (Figure 3B,D–J). Nevertheless, the classic plant-sourced ALA had almost no effect on Aβ-induced neurotoxicity (Figure 3B), though it shows the similar but less-extent effect with other ω-3 fatty acids in the regulation of some markers (Figure 3E,F,J). This is consistent with its relatively lower conversion to EPA compared to SDA. These findings highlight the neuroprotective effect of SDA in preventing Aβ-induced mitochondrial dysfunction and neuronal death in vitro.

### 3.3. SDA Protects against Aβ-Induced Oxidative Stress in Rat Hippocampal Cells

**Figure 4:**
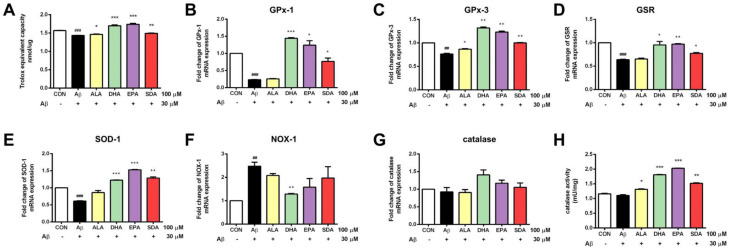
SDA protects against Aβ-induced oxidative stress in rat hippocampal cells. H19-7 cells were pretreated with ALA, DHA, EPA, SDA or BSA–ethanol vehicle control in the concentration of 100 μM for 48 h, then 30 μM Aβ1-40 was added into the medium for another 24 h incubation. (**A**) Total Anti-Oxidant Capacity, mRNA expression of anti-oxidant gene catalase (**B**) GPx-1, (**C**) GPx-3, (**D**) GSR, (**E**) SOD-1, (**G**) catalase and pro-oxidant gene, (**F**) NOX-1 and (**H**) catalase activity in H19-7 cells induced by Aβ1-40. Values were obtained from three independent experiments and were expressed as the means ± SEM; ## *p* < 0.01, ### *p* < 0.001, different from BSA–ethanol vehicle-treated control cells; * *p* < 0.05, ** *p* < 0.01, *** *p* < 0.001, different from Aβ1-40-treated cells.

Mitochondrial dysfunction triggers the production of reactive oxygen species, which increase the oxidative stress in neurons. Evidence indicates that Aβ-induced neuronal cell toxicity is mediated through the excessive oxidative stress. ω-3 fatty acids are natural anti-oxidants and can increase cellular antioxidant capacity in many pathogenic conditions [32]. To determine the effect of SDA on the anti-oxidant defense system of Aβ-induced H19-7 cells, intracellular total anti-oxidant capacity (T-AOC) was measured. As shown in Figure 4A, H19-7 cells treated with Aβ exhibited decreased T-AOC. Enzymatic antioxidants including glutathione peroxidase 1 (Figure 4B), glutathione peroxidase 3 (Figure 4C), glutathione reductase (Figure 4D), and superoxide dismutase (Figure 4E) were decreased in the Aβ-treated group when compared to the control group. Aβ treatment also increased the expression of pro-oxidative gene NADPH oxidase (Figure 4F). ω-3 fatty acid treatment restored the T-AOC (Figure 4A), up-regulated the gene expression of antioxidant enzymes (Figure 4B–E) and inhibited the expression of pro-oxidant enzyme (Figure 4F) in Aβ-induced H19-7 cells. Although Aβ could not change neither the expression of anti-oxidant enzyme catalase (Figure 4G) nor the enzymatic activity of catalase (Figure 4H), pretreatments of ω-3 fatty acids were found to significantly improve the enzymatic activity of catalase compared with the Aβ-induced cells (Figure 4H), which contributed to the overall improvement in T-AOC of H19-7 cells (Figure 4A). Again, we found that the protective effect of SDA on Aβ-induced oxidative stress was comparable to that of EPA and DHA, but much more effective than that of ALA (Figure 4). These findings highlight the neuroprotective effect of SDA in preventing Aβ-induced oxidative stress in vitro.

### 3.4. SDA Protects against Aβ-Induced Inflammation in Rat Hippocampal Cells

The co-occurrence of mitochondrial dysfunction–oxidative stress and neuroinflammation are alleged pathogenic mechanisms of neuronal degeneration [33]. Based on various AD mouse models, it is known that higher levels of cytokines trigger inflammation and thereby exacerbate AD pathology [34]. In order to evaluate the effect of SDA on Aβ-induced neuroinflammation in hippocampal cells, the levels of pro-inflammatory cytokines including IL-1β, IL-6, and TNFα were evaluated by ELISA. However, treatment of Aβ40 alone in H19-7 cells for 24 h was not able to induce the release of these proinflammatory cytokines (data not shown), suggesting that the Aβ-induced neuroinflammation in hippocampus in vivo was through Aβ’s activation on hippocampal microglial cells, which release the production of cytokines [35], but not through a direct effect on hippocampal neurons. LPS is another commonly used mediator to induce inflammatory processes in vitro and it was shown to induce the production of pro-inflammatory cytokines from neurons [36]. Therefore, LPS was added together with Aβ to induce hippocampal neuronal inflammation in the present study.

**Figure 5:**
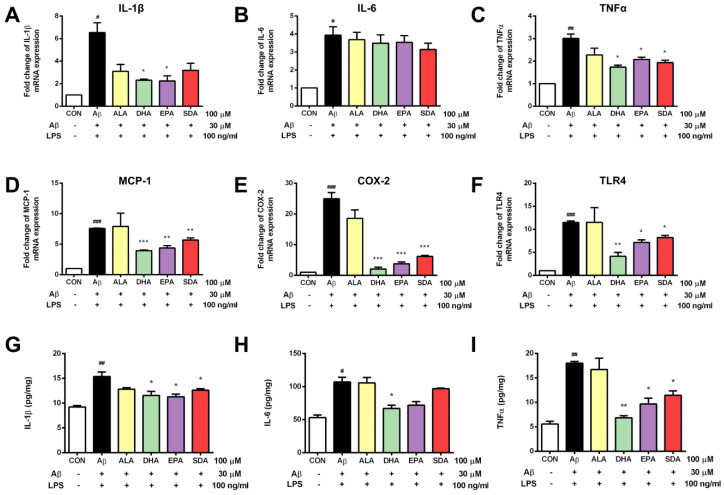
SDA protects against Aβ-induced inflammation in rat hippocampal cells. H19-7 cells were pretreated with ALA, DHA, EPA, SDA, or BSA–ethanol vehicle control in the concentration of 100 μM for 48 h, then 30 μM Aβ1-40 and 100 ng/mL LPS were added into the medium for another 24 h incubation. The release of pro-inflammatory cytokines (**A**) IL-1β, (**B**) IL-6, and (**C**) TNFα and the mRNA expression of proinflammatory mediators (**D**) IL-1β, (**E**) IL-6, (**F**) TNFα, (**G**) MCP-1, (**H**) COX-2, and (**I**) TLR4 in H19-7 cells induced by Aβ1-40 and LPS. Values were obtained from three independent experiments and were expressed as the means ± SEM. # *p* < 0.05, ## *p* < 0.01, ### *p* < 0.001, different from BSA–ethanol vehicle-treated control cells; * *p* < 0.05, ** *p* < 0.01, *** *p* < 0.001, different from Aβ- and LPS-treated cells.

As shown in Figure 5, H19-7 cells treated with Aβ and LPS exhibited increased hippocampal neuroinflammation. The gene expression of pro-inflammatory cytokines including IL-1β (Figure 5A), IL-6 (Figure 5B), and TNFα (Figure 5C) was increased in the Aβ- and LPS-treated group when compared to the control group. Aβ and LPS treatment also increased the expression of inflammatory markers, including MCP-1 (Figure 5D), COX-2 (Figure 5E), and TLR4 (Figure 5F). The levels of secreted cytokines released into culture media were determined by ELISA. Consistent with gene expression levels, the protein levels of pro-inflammatory cytokines were also significantly increased by Aβ and LPS induction (Figure 5G–I). ω-3 fatty acid pretreatment attenuated the Aβ- and LPS-induced inflammatory response in H19-7 cells. Specifically, SDA, EPA, and DHA effectively inhibited the gene expression of MCP-1 (Figure 5D), COX-2 (Figure 5E), and TLR4 (Figure 5F), as well as attenuated the release of IL-1β (Figure 5A,G) and TNFα (Figure 5C,I) induced by Aβ and LPS. The induced production of IL-6 was only changed by DHA pretreatment (Figure 5H). Again, we found that the protective effect of SDA on Aβ- and LPS-induced neuroinflammation was comparable to that of EPA and DHA, but much more effective than that of ALA (Figure 5). It is worth to mention that ALA was found to have no effect on Aβ- and LPS-induced neuroinflammation (Figure 5) while it might have a small effect on Aβ-induced apoptosis (Figure 3) and oxidative stress (Figure 4). These findings highlight the neuroprotective effect of SDA in preventing Aβ- and LPS-induced inflammatory response in vitro.

### 3.5. The Anti-Aβ Effect of SDA Involves Its Inhibition on APP Expression and Regulation on MAPK Signaling

**Figure 6:**
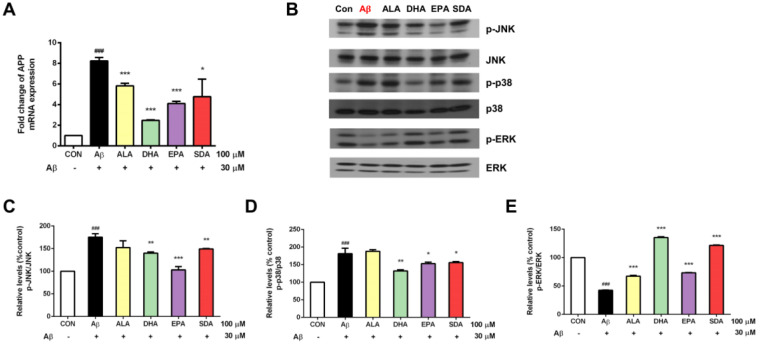
The anti-Aβ effect of SDA involves its inhibition on Aβ synthesis and regulation on MAPK signaling. H19-7 cells were pretreated with ALA, DHA, EPA, SDA, or BSA–ethanol vehicle control in the concentration of 100 μM for 48 h, then 30 μM Aβ1-40 was added into the medium for another 24 h incubation. (**A**) The mRNA expression of APP. (**B**) Western blot analysis of MAPK signaling activation. The ratios of (**C**) p-JNK to JNK, (**D**) p-p38 to p38 and (**E**) p-ERK to ERK were determined. Values were obtained from three independent experiments and were expressed as the means ± SEM. ### *p* < 0.001, different from BSA–ethanol vehicle-treated control cells; * *p* < 0.05, ** *p* < 0.01, *** *p* < 0.001, different from Aβ-treated cells.

Increased oxidative stress and release of proinflammatory mediators were found to induce the production of Aβ precursor protein (APP) [37]. APP is the precursor molecule whose proteolysis generates Aβ, the primary component of amyloid plaques found in the brains of AD patients. Since Aβ-induced oxidative damage was seen in our experiments (Figure 4), we then checked if Aβ would further induce APP gene expression. As shown in Figure 6A, the exposure of H19-7 neurons to 30 μM Aβ40 for 24 h resulted in a significant increase in the gene expression of APP compared to the untreated control. Subsequently, we hypothesized that the protective effect of SDA on Aβ-induced neurotoxicity was through its inhibition on APP gene expression. Indeed, we found that H19-7 cells pretreated with ω-3 fatty acids (ALA, DHA, EPA, and SDA) prior to Aβ40 significantly down-regulated the mRNA level of APP compared with the Aβ-treated cells (Figure 6A). As an integral membrane protein in neurons, APP functions not only as the precursor of Aβ, but also a cell surface receptor which has been implicated to promote transcriptional activation [38], has antimicrobial activity [39], regulates synapse formation [40] and neural plasticity [41], is involved in copper-related oxidative stress and neuronal death [42] as well as induces the activation of p38 MAPK, leading to the internalization of Aβ and mitochondrial dysfunction [43]. These physiological and pathological processes are all linked to MAPK pathways which relay, amplify and integrate signals from a diverse range of stimuli and elicit an appropriate response including cellular proliferation, differentiation, development, inflammatory responses and apoptosis in mammalian cells [44]. In fact, MAPK pathways were often found to mediate oligomeric Aβ-induced neurotoxicity [45,46]. In the present study, we found that the exposure of H19-7 neurons to 30 μM Aβ40 for 24 h significantly increased JNK (Figure 6B,C) and p38 (Figure 6B,D) phosphorylation and decreased ERK (Figure 6B,E) phosphorylation compared to the untreated control. Based on these results, we hypothesized that the protective effect of SDA on Aβ-induced neurotoxicity occured through its regulation on MAPK signaling. Indeed, we found that H19-7 cells pretreated with ω-3 fatty acids prior to Aβ significantly reduced the activation of JNK (Figure 6B,C) and p38 (Figure 6B,D), and increased the activation of ERK (Figure 6B,E) compared with the Aβ-treated cells. Consistently, the protective effect of SDA on Aβ-induced damage was comparable to that of EPA and DHA, but much more effective than that of ALA (Figure 6). These findings highlight the anti-Aβ effect of SDA involves its inhibition on APP gene expression and regulation on MAPK pathways.

## 4. Discussion

The World Health Organization (WHO), as well as many other authorities, recommends consumption of oily fish once or twice a week in order to ensure dietary intake of ω-3 PUFAs with recognized health benefits [47,48,49,50]. Biologically active ω-3 PUFAs generally include EPA and DHA, which are derived from the ocean. In recent years, an increased interest in research is plant-sourced ω-3 fatty acids, ALA and SDA, which are often converted into EPA and DHA for health benefits. ALA is the metabolic precursor of EPA and DHA (Figure 1), but the conversion of ALA to EPA and DHA is very limited due to the low enzymatic activity of Δ6 desaturase in humans. SDA, an intermediate in the pathway of EPA and DHA biosynthesis, is the product of ALA desaturation by Δ6 desaturase which is more readily converted to EPA and appears to offer better potential for health improvement than ALA due to skipping rate-limiting enzymes [8]. The ameliorating effect of dietary ω-3 fatty acids on AD has been recognized. Epidemiological studies indicated that increased consumption of DHA and EPA from fish oil or fatty fish are associated with reduced risk of AD [51]. For instance, van Gelder and colleagues examined cognitive decline over a 5-year period and reported that increase in fish consumption and DHA/EPA intake are both associated with reduction in cognitive decline [52]. In studies of transgenic AD mouse models or aged animals, learning, reference and working memory performance was also found to be enhanced by supplementation with ω-3 fatty acids [53,54,55]. Furthermore, the contents of ω-3 fatty acids have been reported to be significantly decreased in the plasma and brain of patients with AD as compared to healthy controls, suggesting a possible role of ω-3 fatty acids in the intervention of AD [56]. However, the specific molecular mechanism is not clear. In this research, we have demonstrated for the first time that SDA can be efficiently converted to EPA in the hippocampus, which is the cognitive and memory center. This wonderfully expands what we know about the health benefits of SDA.

The genetic and biosynthetic machinery for elongation of ω-3 fatty acid precursors is expressed in hippocampal cells (including cell lines), yet at much lower levels than in lipogenic tissues [57], suggesting the final steps of elongation, desaturation and final beta oxidation to DHA might operate with relatively slow kinetics in hippocampus. This might explain our finding indicating no EPA to DHA conversion (Figure 2B). EPA and DHA are important structural components of brain cells and have a crucial impact on brain function [58]. DHA is the most abundant ω-3 fatty acid in cell membranes, which varies greatly in various organs, and is especially abundant in nervous tissues such as the brain and retina where the content reaches 14 g/100 g and 22 g/100 g of total fatty acids, respectively. Our results showed that the untreated hippocampal cells contain about 30 ng/mg of EPA and 4 ng/mg of DHA (Figure 2). Polyunsaturated fatty acids and their bioactive derivatives have been shown to regulate neurogenesis and brain inflammation. Moreover, the altered fatty acid signaling in brain has been linked to mood disorders, cognition, Alzheimer’s disease, schizophrenia and other disorders [59]. Hippocampal formation is important for memory [60]. In rats, the performance of spatial memory has been found to be tightly associated with hippocampal activity [61]. The apoptosis of hippocampal neurons played a key role in the learning and memory deficit [62]. The above studies show that EPA and DHA in hippocampal cells are essential for brain functions such as cognition and memory, which has a positive effect on the treatment of AD.

Aβ40 together with Aβ42 are two major C-terminal variants of the Aβ protein constituting the majority of Aβs. These undergo post-secretory aggregation and deposition in the Alzheimer’s disease brain [63]. While it is generally agreed that unmutated Aβ42 is much more toxic than unmutated Aβ40, the concentration of Aβ40 in cerebral spinal fluid has been found to be several-fold more than that of Aβ42 [64]. In the early stage, Aβ40 exists in rapid equilibrium in the form of monomers, dimers, trimers and tetramers, while Aβ42 can form stable pentamers and hexamers [65]. Correspondingly, it was found that the molecular cycle of Aβ40 fibrils is faster than that of Aβ42 fibrils. Further studies showed that Aβ40 has a higher rate constant for the separation of molecules from fibers than Aβ42 [66]. Moreover, both Aβs promoted neural progenitor cell (PCs) growth and neurogenesis in mice. However, soluble Aβ40 can induce NPCs to differentiate into neurons, while Aβ42 induces NPCs to differentiate into astrocytes [67]. Particularly, Aβ40 was found to inhibit ex vivo hippocampal revascularization and therefore involved in the initial progression of AD [68]. Therefore, Aβ40 was used in our study to induce hippocampal AD-like damage. 

DHA and EPA have been reported to prevent oxidative stress in cultured neurons associated with AD [69]. In the present study, NOX-1 expression induced by Aβ was significantly reduced by DHA. Pretreatment of EPA and SDA did not significantly affect NOX-1 expression. This is consistent with our results that SDA could effectively convert to EPA, but not DHA in H19-7 cells, and suggesting that DHA may have its unique mechanism in mediating neuroprotective effects. Catalase expression was not significantly affected by any ω-3 fatty acid treatment, but the activity of catalase was significantly improved by pretreatment of DHA, EPA, and SDA. The expression of GPx-1, GPx-3, GSR, and SOD-1 depressed by Aβ was all significantly increased by pretreatment of DHA, EPA, and SDA. GPx-3 is a plasma glutathione peroxidase that is synthesized intracellularly and secreted extracellularly. Our results found that ω-3 fatty acid can increase GPX-3 gene expression in hippocampal cells, suggesting that ω-3 fatty acid may also play a role in extracellular oxidative stress, which requires further studies. We can even explore whether ω-3 fatty acid can lead to increased GPX3 protein secretion in vivo. In addition, the antioxidant enzyme GPx-4 is the most important glutathione peroxidase in preventing oxidative damage of nerve cell membranes. DHA can enhance the transcriptional activity of GPx-4 and thus enhance the antioxidant capacity of hippocampal cells [70]. In our study, we detected the expression of GSR, an efficient enzyme downstream of GPX-4, in the experiment, which also explained the role of GPX-4 to some extent.

Aβ normally induces hippocampal inflammation by activating microglia [71,72]. Specifically, Aβ accumulation increases the production of pro-inflammatory cytokines such as IL-1β and TNFα from microglial. Few studies have investigated the direct induction of neuron inflammation by Aβ. Actually, we found that treatment of Aβ40 alone in H19-7 cells for 24 h was not able to induce the release of these proinflammatory cytokines. LPS is another commonly used mediator to induce inflammatory processes in vitro and it was shown to induce the production of pro-inflammatory cytokines from neurons [73]. LPS triggers an array of microglial response by interacting with the membrane receptor Toll-like receptor 4 (TLR4), leading to the production of pro-inflammatory mediates and the self-activation of the nuclear factor-κB system [74]. Previous studies reported increased TLR4 expression and inflammatory cytokine release in neurons when exposed to Aβ [75]. We also found that H19-7 cells secreted pro-inflammatory cytokines after LPS treatment. In previous studies, 1 mg/mL LPS was commonly used to induce neuronal inflammation [36]. In our study, 100 ng/mL LPS together with Aβ could induce a significant inflammatory response, suggesting that LPS and Aβ may have a synergistic effect. In fact, LPS has been shown to significantly increase Aβ accumulation [76]. 

The mechanism underlying Aβ-induced neurotoxicity is complex, involving several signaling pathways. Recent evidence suggests that Aβ could stimulate JNK and p38 activation, which might be involved in AD pathogenesis [77,78,79]. It remains controversial for Aβ-induced ERK signaling. Some studies showed activation of ERK phosphorylation [78,80], some demonstrated inhibition of ERK phosphorylation [69], while some found stable phosphorylation profile of ERK after Aβ treatment [77]. Here, we found that phosphorylated JNK and p38 were markedly increased, while phosphorylated ERK was dramatically decreased after Aβ40 treatment. Generally, phosphorylation of JNK and p38 is highly activated in response to a variety of stress signals, including oxidative stress and proinflammatory cytokines, while the activation of ERK pathway promotes cell growth, differentiation and survival [81]. Previous studies demonstrated that DHA pretreatment significantly increases neuronal survival upon Aβ treatment by promoting ERK-related survival pathway [69]. ERK inhibitor, U0126, abolished DHA-induced ERK phosphorylation and neurogenesis in human neuronal cells [82]. Here, we found that DHA, EPA, and SDA were all able to block the activation of JNK/p38 phosphorylation induced by Aβ and meanwhile improve ERK phosphorylation depressed by Aβ. ERK activity is mediated by Ras, but the activities of JNK and p38 are Ras-independent [83], suggesting that the effects of fatty acids are associated with both Ras-dependent and Ras-independent pathways. In the present study, we demonstrate that SDA exerts its neuroprotective properties against Aβ by inhibiting stress JNK/p38 signaling and improving ERK survival signaling. Future experiments are suggested to identify the upstream anti-apoptotic pathways triggered by ω-3 fatty acids in neurons.

In future studies, we need to verify the function of SDA in vivo models. Although H19-7 cell line is widely used as a model to study Alzheimer’s disease, there are differences between real hippocampal cells and hippocampal cell lines. Martin et al. (2006) found that the lipid composition of cell membrane in brain cell lines was significantly different from that of brain tissue [84]. The mechanism by which the brain absorbs polyunsaturated fatty acids is not well understood and remains controversial. However, because most polyunsaturated fatty acids come from diet, changes in their intake can alter the level of polyunsaturated fatty acids in the brain [59]. Although the ability of SDA to cross the blood–brain barrier was not studied, both EPA and DHA can cross the blood–brain barrier [85,86], so, presumably, SDA should be able to cross the blood–brain barrier, too. However, this needs to be confirmed in future studies.

## 5. Conclusions

**Figure 7:**
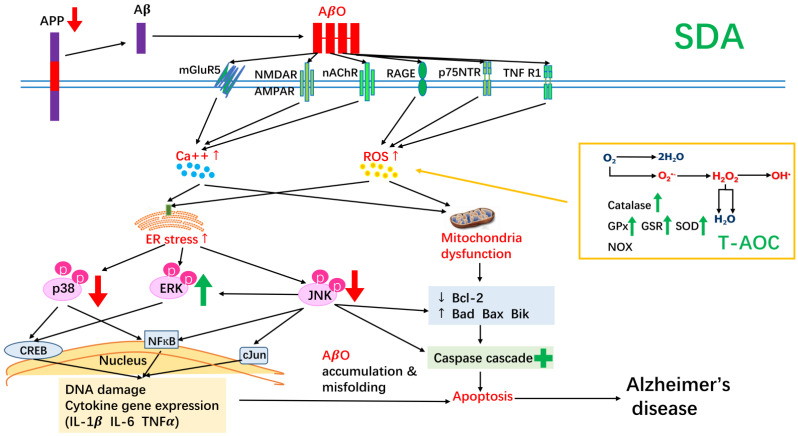
A summary of the neuroprotective effect of SDA on amyloid β-induced neurotoxicity in rat hippocampal cells. SDA was able to regulate expression of apoptotic mediators, improve total anti-oxidant capacity, reduce expression of pro-inflammatory mediators, inhibit expression of the precursor of Aβ, attenuate stress-triggered apoptotic JNK/p38 phosphorylation, and activate survival-related ERK signaling pathway.

In summary, our results suggest that the ω-3 fatty acid, SDA, provides hippocampal neurons with a higher resistance level to the cytotoxic effects induced by Aβ. Specifically, SDA effectively converted to EPA in hippocampal cells (17% as effective as EPA, 1.5-fold more than ALA). SDA was able to regulate expression of apoptotic mediators, improve total anti-oxidant capacity, reduce expression of pro-inflammatory mediators, inhibit expression of the precursor of Aβ, attenuate stress-triggered apoptotic JNK/p38 phosphorylation, and activate survival-related ERK signaling pathway (Figure 7). This is the first time that such protective properties have been reported with SDA. A diet rich in ω-3 fatty acids may therefore reduce Aβ-mediated cytotoxicity, neuronal loss and the risk of developing AD.

## Figures and Tables

**Table 1:** Oligonucleotide primer sequences used in real-time PCR.

Gene	Forward Primer	Reverse Primer	Accession Number
APP	5′-TCAGATTGCGATGTTCTGTGG-3′	5′-CTGGCTGGTTTGCTTCCATCA-3′	NM_019288.2
Bad	5′-AAGTCCGATCCCGGAATCC-3′	5′-GCTCACTCGGCTCAAACTCT-3′	NM_022698.2
Bax	5′-TGAAGACAGGGGCCTTTTTG-3′	5′-AATTCGCCGGAGACACTCG-3′	NM_017059.2
Bcl-2	5′-GTCGCTACCGTCGTGACTTC-3′	5′-CAGACATGCACCTACCCAGC-3′	NM_016993.2
Bik	5′-ACTGTTCCACACGACCAGG-3′	5′-CACAGGACTAAGGTTTTCCCC-3′	NM_053704.2
Caspase-3	5′-ATGGAGAACAACAAAACCTCAGT-3′	5′-TTGCTCCCATGTATGGTCTTTAC-3′	NM_012922.2
Catalase	5′-AGCGACCAGATGAAGCAGTG-3′	5′-TCCGCTCTCTGTCAAAGTGTG-3′	NM_012520.2
COX-2	5′-TGAGCAACTATTCCAAACCAGC-3′	5′-GCACGTAGTCTTCGATCACTATC-3′	NM_017232.4
GPx-1	5′-AGTCCACCGTGTATGCCTTCT-3′	5′-GAGACGCGACATTCTCAATGA-3′	NM_030826.4
GPx-3	5′-TCACACTTTCTCCAGGTTCCCGTT-3′	5′-TCATGTGGGCATATGGGAGATGCT-3′	NM_022525.4
GSR	5′-GACACCTCTTCCTTCGACTACC-3′	5′-CCCAGCTTGTGACTCTCCAC-3′	NM_053906.2
IL-1β	5′-GCAACTGTTCCTGAACTCAACT-3′	5′-ATCTTTTGGGGTCCGTCAACT-3′	NM_031512.2
IL-6	5′-TAGTCCTTCCTACCCCAATTTCC-3′	5′-TTGGTCCTTAGCCACTCCTTC-3′	NM_012589.2
MCP-1	5′-TTAAAAACCTGGATCGGAACCAA-3′	5′-GCATTAGCTTCAGATTTACGGGT-3′	NM_031530.1
NOX-1	5′-GGTTGGGGCTGAACATTTTTC-3′	5′-TCGACACACAGGAATCAGGAT-3′	NM_053683.2
SOD-1	5′-AACCAGTTGTGTTGTCAGGAC-3′	5′-CCACCATGTTTCTTAGAGTGAGG-3′	NM_017050.1
TLR4	5′-GCCTTTCAGGGAATTAAGCTCC-3′	5′-AGATCAACCGATGGACGTGTAA-3′	NM_019178.2
TNFα	5′-CCCTCACACTCAGATCATCTTCT-3′	5′-GCTACGACGTGGGCTACAG-3′	NM_012675.3
β-actin	5′-GGCTGTATTCCCCTCCATCG -3′	5′-CCAGTTGGTAACAATGCCATGT-3′	NM_031144.3

## Data Availability

Not applicable.
